# Exogenous glycine betaine alleviates dynamic physiological and transcriptomic responses in *Inula salsoloides* under combined salt-cadmium stress

**DOI:** 10.3389/fpls.2026.1760419

**Published:** 2026-01-30

**Authors:** Heyi Wang, Lei Wang, Xiaomin Zhang, Lu Cao, Guiquan Wang, Yunpeng Zhang, Quan Hao, Ze Re, Ruhan A, Zhicheng Zhe, Xiaoyun Yan

**Affiliations:** 1 Forestry College, Inner Mongolia Agricultural University., Hohhot, China; 2 Inner Mongolia Expressway Shanmei Ecological Development Co., Ltd., Hohhot, China

**Keywords:** combined salt-cadmium stress, glycine betaine, *Inula salsoloides*, physiological dynamics, transcriptomics

## Abstract

**Introduction:**

*Inula salsoloides*, a psammophytic and salt-tolerant xerophyte is ecologically valuable for windbreaks, sand fixation, and the restoration of saline-alkali lands. However, the regulatory mechanisms through which exogenous glycine betaine (GB) alleviates combined salt-cadmium stress in this species remain unclear.

**Methods:**

This study investigated the protective effects of foliar-applied GB on *Inula salsoloides* seedlings under individual salt, cadmium, and combined salt-cadmium stress through a systematic analysis of seed germination, seedling growth, key physiological and biochemical parameters, and transcriptome sequencing.

**Results:**

The salt, cadmium, and combined salt-cadmium stress significantly suppressed seed germination and seedling growth, impaired photosynthetic pigment synthesis, and induced oxidative damage, as reflected by reactive oxygen species accumulation and membrane lipid peroxidation. Exogenous GB application effectively mitigated these adverse effects, increasing germination potential, vigor index, and biomass. Physiologically, GB promoted the accumulation of osmoprotectants and enhanced antioxidant enzyme activities, thereby restoring cellular osmotic and redox homeostasis. Transcriptomic analysis revealed that GB reprogrammed the expression of genes enriched in key metabolic pathways, including carbon fixation in photosynthetic organisms, glyoxylate and dicarboxylate metabolism, and particularly, arginine and proline metabolism under combined stress. GB also modulated the expression of numerous stress-related transcription factors.

**Discussion:**

Our findings demonstrate that exogenous GB enhances the tolerance of *Inula salsoloides* to salt and cadmium stress by coordinating physiological and transcriptional responses. This study provides novel insights into the mechanisms of GB-mediated stress alleviation and supports its potential application in enhancing plant ecological resilience in saline and heavy metal-contaminated environments.

## 1 Introduction

Soil salinization is a pervasive global environmental issue that poses a persistent threat to agricultural and natural ecosystems (Shabala, 2013). Combined salt-alkali stress generally inflicts more severe damage on plants compared to individual salt stress. The detrimental effects include exacerbated ion imbalance, specific ion toxicity, impaired osmotic adjustment, and suppression of antioxidant enzyme activities. These factors collectively and synergistically inhibit plant growth and development (Chen et al., 2017; Wang Y, et al., 2020; Xu et al., 2025). Under high salinity conditions, elevated soil osmotic potential induces osmotic stress, resulting in a physiological condition akin to drought stress within plant cells (Hoque et al., 2022). In response to water deficit, plants partially close their stomata; however, this consequently restricts carbon dioxide (CO_2_) uptake and diffusion, thereby inhibiting critical photosynthetic processes such as photophosphorylation and ultimately compromising photosynthetic capacity (Khalid et al., 2015). This multi-layered stress response mechanism has been documented across diverse crop species, underscoring a conserved adaptation pathway to salt stress among plants (Rahnama et al., 2010).

Saline-alkali stress primarily affects plants through root osmotic stress, ion toxicity, and nutrient deprivation (Tahjib-Ul-Arif et al., 2023). These factors contribute to diminished root activity, impaired translocation of water and nutrients, and reduced efficiency in internal assimilate partitioning. Consequently, plants often exhibit stunted growth or may even succumb to carbon starvation (Liu et al., 2025; Li X, et al., 2025). Simultaneously, such stress disrupts intracellular redox homeostasis, resulting in substantial accumulation of reactive oxygen species (ROS). Excessive ROS, in turn, inhibits the activities of key enzymes, diminishes photosynthetic efficiency, induces organelle damage, and accelerates plant senescence (Miller et al., 2010; Zahra et al., 2022).

Furthermore, the long-term accumulation of heavy metals in soils can profoundly alter their physicochemical properties and compromise the stability and functioning of soil ecosystems (Ollivier et al., 2012; Shang et al., 2024). Among these metals, cadmium (Cd), a non-essential and highly phytotoxic element, exhibits significant mobility and a strong potential for bioaccumulation. Once Cd concentration in plant tissues exceeds a critical threshold, it elicits multifaceted toxicity. This not only directly impedes normal plant growth and development (Clemens, 2006) but also poses substantial risks to public health through its transfer along the food chain, ultimately endangering animal and human health (Satarug et al., 2010). At the physiological level, elevated Cd exposure systemically disrupts various vital plant processes. These disruptions include the inhibition of photosynthetic pigment biosynthesis and photosystem function (Cao et al., 2017), disruption of antioxidant enzyme systems and the activities of enzymes involved in nitrogen metabolism (Yang et al., 2019), impediment of cell division and elongation (Liu et al., 2015), and compromise of plasma membrane integrity (Yue and Yang, 2015). Cd stress also adversely affects fundamental metabolic processes such as respiration and transpiration. Collectively, these disturbances lead to a severe suppression of overall plant growth and development.

*Inula salsoloides* (*I. salsoloides*) is a perennial herb of the *Asteraceae* family, widely distributed across desert, gobi, arid steppe, and saline-alkaline wasteland habitats in northern and northwestern China. It is recognized as a typical psammophytic and salt-tolerant xerophyte. The species is characterized by a low-growing, multi-branched architecture, with succulent leaves covered with gray-white pubescence, and a well-developed root system—key morphological adaptations to arid and highly saline environments (Wang et al., 2024). *I. salsoloides* also possesses a prolonged flowering period, vibrant yellow inflorescences, and an attractive form, endowing it with both ornamental and ecological value for use in landscape gardening and ecological restoration (Zhao, 2012). Ecologically, *I. salsoloides* is considered a pioneer species and is widely used in windbreaks, sand stabilization, and the remediation of saline-alkaline soils (Niu et al., 2018).

Its seed germination and seedling growth exhibit considerable tolerance to fluctuations in temperature, water availability, and salt stress (Li D, et al., 2025). However, in natural habitats, the species often faces more complex, combined stresses such as simultaneous salt and Cd stress, which can significantly inhibit seed germination and seedling establishment (Du, 2010; Spiridonova et al., 2019; Zia-ur-Rehman et al., 2023). These combined stresses not only impede root development and induce leaf chlorosis and wilting (Munns and Tester, 2008; Shi et al., 2021) but also trigger a series of physiological and biochemical disruptions at the cellular and molecular levels, adversely affecting normal morphogenesis and metabolic functions (Liu et al., 2020; Yang, 2020; Zhao et al., 2014). To understand these detrimental effects, it is useful to consider the primary mechanisms of each stressor individually. Studies indicate that salt stress primarily inhibits plant growth through pathways such as osmotic stress, ion toxicity, and oxidative stress (Yang and Guo, 2018), whereas Cd stress impacts plant physiology by disrupting photosynthesis, inhibiting enzymatic activity, and disrupting cellular structures (An et al., 2019; Clemens, 2006). The negative impacts of such abiotic stresses can be mitigated through biotic interactions. Notably, microbial interactions can significantly enhance the stress resistance of *I. salsoloides*. For instance, arbuscular mycorrhizal fungi (AMF) have been shown to improve its growth and physiological adaptation under drought stress by expanding the root absorption area, enhancing antioxidant capacity, and regulating osmolyte content (Li et al., 2013). Given this combination of inherent tolerance, sensitivity to complex stresses, and modifiable resilience, *I. salsoloides* serves not only as a key species in desertification control and ecological rehabilitation but also as an ideal model organism for investigating multifaceted plant stress resistance mechanisms.

In recent years, the application of exogenous plant growth regulators to enhance stress resistance by modulating internal physiological and biochemical processes has garnered increasing interest. Glycine betaine (GB), a quaternary ammonium compound, is a non-toxic osmolyte widely found in microorganisms, plants, and animals, and possesses relatively stable physicochemical properties. Under abiotic stresses such as drought, salinity, and low temperature, GB functions to regulate cellular osmotic pressure, stabilize the structure and function of cell membranes and macromolecules (Chen and Murata, 2011), protect the photosynthetic system (Ashraf and Foolad, 2005), and scavenge ROS (Islam et al., 2009; Ma et al., 2016). GB has been shown to improve seedling tolerance to various stresses, including drought, salinity, and heavy metals (Huang and Yang, 2017; Hua, 2022; Lu et al., 2019). Studies indicate that foliar application of GB at the seedling stage effectively enhances plant stress resistance, mitigating damage from adverse conditions (Gao et al., 2011), improving drought resistance (Hou et al., 2013), and alleviating the detrimental effects of abiotic stress on seedlings (Zhao et al., 2020). The mechanisms by which GB enhances plant abiotic stress tolerance are multifaceted, involving the regulation of plant growth and development, cellular osmotic adjustment, membrane stability, the antioxidant system, and gene expression (Annunziata et al., 2019; Rathinasabapathi et al., 2001).

Despite considerable progress in understanding GB-mediated stress tolerance, its physiological roles and underlying mechanisms in *I. salsoloides* remain poorly understood. Systematic investigations into the physiological regulation of exogenous GB in *I. salsoloides* under individual salt (S), Cd, and combined salt-cadmium (SCd) stress are scarce, and the transcriptional responses are particularly unclear. To address this knowledge gap, we applied exogenous GB via foliar spraying to systematically assess changes in seed germination and physiological parameters of *I. salsoloides* under S, Cd, and SCd stress. Combined with transcriptome sequencing, we aimed to identify key differentially expressed genes (DEGs) and enriched metabolic pathways regulated by GB. This study provides new insights into the mechanisms of GB-enhanced stress resistance and establishes a theoretical foundation for using GB to improve plant resilience and ecological restoration in salinized and heavy metal-contaminated environments.

## 2 Materials and methods

### 2.1 Experimental design and plant materials

This experiment was conducted from May to August 2025 at the Forestry College, Inner Mongolia Agricultural University (111°41′E-111°37′E, 40°48′N-40°68′N). Seeds of *I. salsoloides* were collected from wild populations in Wuhai City, Inner Mongolia (106°48′E-109°31′E, 38°45′N-40°45′N) in July 2024. After natural air-drying, seeds were stored at 4°C temperature until use. Preliminary cultivation experiments were performed in the intelligent greenhouse and the forest tissue culture room of the Forestry College. Physiological measurements were conducted in the Garden Plant Laboratory of the same college.

### 2.2 Growth conditions and stress treatments

Plants were grown under controlled environmental conditions: temperature 25 ± 3°C, relative humidity 50%–60%, 12 h light/12 h dark photoperiod, and light intensity of 6000 Lux. One day before sowing, seeds were surface-sterilized with 5% sodium hypochlorite for 5 minutes, followed by three rinses with distilled water. Sterilized seeds were then soaked in various treatment solutions for 24h in darkness at room temperature to remove non-viable seeds. Sunken seeds were selected for germination assays.

The experiment included eight treatment groups: CK (distilled water control), GB (50 mmol/L GB), S (50 mmol/L Na_2_CO_3_:NaHCO_3_ = 1:1), SGB (50 mmol/L GB + 50 mmol/L Na_2_CO_3_:NaHCO_3_ = 1:1), Cd (150 μmol/L CdCl_2_), CdGB (150 μmol/L CdCl_2_ + 50 mmol/L GB), SCd (50 mmol/L Na_2_CO_3_:NaHCO_3_ = 1:1 + 150 μmol/L CdCl_2_), and SCdGB (50 mmol/L Na_2_CO_3_:NaHCO_3_ = 1:1 + 150 μmol/L CdCl_2_ + 50 mmol/L GB). The concentrations for salt (50 mmol/L Na_2_CO_3_:NaHCO_3_ = 1:1), Cd (150 μmol/L CdCl_2_), and GB (50 mmol/L GB) were determined through preliminary phenotypic screening. Uniform seedlings were exposed to gradients of each stressor, and concentrations inducing clear but sub-lethal growth inhibition were selected for the main experiment. GB concentration was similarly optimized, with 50 mmol/L showing the most consistent mitigation of these predefined stress symptoms. Each treatment consisted of 500 seeds with three biological replicates. We sowed seeds in round plastic trays (12 cm length × 10 cm width × 7 cm height) filled with a sterilized substrate mixture consisting of 30% white peat moss and 70% black peat moss (both sphagnum-based). Germination was monitored daily for 42 days, with the experiment terminating after seven consecutive days without new germination. At the seedling stage, plants were foliar-sprayed with corresponding treatment solutions every three days. This included spraying the GB group with 50 mmol/L GB, allowing us to assess its direct growth-promoting effect separately from its stress-alleviating role. The GB concentration for foliar spray was consistent with that used for seed soaking (50 mmol/L GB). Spraying continued until the day before sample harvest. Uniformly grown plants were harvested 24 hours after the final stress treatment application for subsequent physiological and transcriptomic analyses, corresponding to the peak period of stress symptom expression.

### 2.3 Measured parameters and analytical methods

#### 2.3.1 Seed germination parameters

In the germination assay, seeds were monitored daily starting 24 h after sowing (designated as Day 1). The assay concluded after seven consecutive days without new germination, which occurred on Day 14 in this experiment. Germination potential (GP), germination rate (GR), germination index (GI), and vigor index (VI) were then calculated. GP and GR were determined following International Seed Testing Association (ISTA) standards. GI and VI were calculated according to established methods for seed vigor assessment (Bewley et al., 2013; Coolbear et al., 1984), using the following formulas (Equations 1–4):

(1) $$GP=\frac{Number\;of\;seeds\;germinated\;by\;day\;6}{Total\;seeds}\times 100\%$$

(2) $$GR=\frac{Number\;of\;seeds\;germinated\;by\;day\;14}{Total\;seeds}\times 100\%$$

(3) $$GI=\sum \frac{Gt}{Dt}$$

(4) VI= GI×RL

Gt is the number of seeds germinated on day t, Dt is the number of germination days, and RL is the radicle length.

#### 2.3.2 Seedling morphological parameters

At the end of the experiment, ten intact seedlings per treatment were randomly harvested. The roots were gently rinsed to remove adhering soil and blotted dry with filter paper. Morphological parameters, including root length, plant height, leaf length, leaf width, and leaf thickness, were measured using a vernier caliper. For leaf dimensions, the first ten fully expanded leaves from the apex were measured. All measurements were conducted with three biological replicates, and the mean values were calculated.

#### 2.3.3 Physiological and biochemical assays

Following the experimental treatments, fresh leaf samples exhibiting uniform growth were randomly collected from each group for physiological analysis. All assays were conducted with three biological replicates. The activities of key antioxidant enzymes were determined according to Zou (2000) as follows: superoxide dismutase (SOD) using the nitroblue tetrazolium (NBT) method, peroxidase (POD) via the guaiacol method, catalase (CAT) by ultraviolet absorption, and ascorbate peroxidase (APX) with a spectrophotometric assay. The concentration of malondialdehyde (MDA), an indicator of lipid peroxidation, was quantified using the thiobarbituric acid method (Zhang et al., 2009). Levels of ROS, including hydrogen peroxide (H_2_O_2_) and the superoxide anion (O_2_^-^), were measured following the procedure described by Shi (2016).

Furthermore, we assessed various osmoregulatory substances and metabolic indicators. Soluble sugar (SS) content was analyzed by anthrone colorimetry, soluble protein (SP) by ultraviolet absorption and root activity by the triphenyltetrazolium chloride (TTC) reduction method, as outlined by Li (2000). Additional parameters, namely ascorbic acid (AsA; spectrophotometrically), total free amino acids (FAA; ninhydrin colorimetry), nitrate reductase (NR; *in vivo* assay), and free proline (PRO; sulfosalicylic acid extraction), were evaluated based on Shi (2016). Plant tissue water content was measured by the gravimetric method, as described by Wang and Huang (2015) with the modifications outlined in Gao and Cai (2018). Polyphenol oxidase (PPO) activity, reduced glutathione (GSH) content, glutathione reductase (GR) activity, and total phenolic (TP) content were analyzed using commercial assay kits.

The contents of chlorophyll a (Chl a), chlorophyll b (Chl b), total chlorophyll (Chl), and carotenoids (Car) were determined according to Gao and Cai (2018). Absorbance of the extracts was measured at 470, 665, and 649 nm, corresponding to the absorption maxima for carotenoids, Chl a, and Chl b in 95% ethanol, respectively. The pigment concentrations were then calculated using standard equations (Equations 5–8). All procedures were conducted under dim light to prevent photodegradation.

(5) $$Chl\;a=13.95A_{665}-6.88A_{649}$$

(6) $$Chl\;b=24.96A_{665}-7.32A_{649}$$

(7) $$Chl=Chl\;a+Chl\;b=6.63A_{665}+18.08A_{649}$$

(8) $$Car=\frac{1000A_{470}-2.05Chl\;a-114.8Chl\;b}{245}$$

Chl a represents the concentration of chlorophyll a; Chl b represents the concentration of chlorophyll b; Chl represents the concentration of total chlorophyll; Car represents the total concentration of carotenoids; A_665_, A_649_, and A_470_ represent the absorbance of the chloroplast pigment extract at wavelengths of 665nm, 649nm, and 470nm.

#### 2.3.4 RNA extraction and transcriptome sequencing

To obtain a representative transcriptomic profile for each treatment, leaves from 10 uniformly grown seedlings were harvested and pooled to form one biological sample. This sampling procedure was repeated independently three times to generate three biological replicates per treatment. Total RNA was isolated from 0.2 g of each pooled leaf samples. This extraction was performed using the R401 (Genepioneer Biotechnologies, Nanjing, China) and TSJ011-100 (Tsingke Biotechnology Co., Ltd., Beijing, China) kits. The concentration and purity of the extracted RNA were assessed using a Nanodrop2000 spectrophotometer. The quality control thresholds for library construction required a total RNA amount ≥1 μg, concentration ≥35 ng/μL, OD260/280 ≥1.8, and OD260/230 ≥1.0, with sufficient total quantity to meet the requirements for triplicate library preparations. After RNA extraction, purification, and library construction, the libraries were subjected to paired-end (PE150) sequencing using the Illumina NovaSeq X Plus platform with Next-Generation Sequencing (NGS) technology. All sequencing procedures were performed by Genepioneer Biotechnologies (Nanjing, China).

#### 2.3.5 qRT-PCR analysis

To validate the RNA-seq results, the expression levels of four randomly selected DEGs were quantified by qRT-PCR using the β-actin gene as an internal reference. The qRT-PCR analysis was performed by Genepioneer Biotechnologies (Nanjing, China). The primer sequences are listed in **Table 1**. The PCR protocol consisted of an initial denaturation at 95°C for 30s, followed by 40 cycles of denaturation at 95°C for 5s and annealing/extension at 60°C for 20s. A melting−curve analysis was then performed from 60°C to 95°C, with fluorescence data collected at every 1°C increment. Three biological replicates were included. The relative expression levels of target genes were calculated using the 2^−ΔΔCt^ method.

**Table 1:** Primers used for the qRT-PCR validation.

Gene ID	Forward primer (5' to 3')	Reverse primer (5' to 3')
ALDH18A1	TTCCAGAGACTGTTGGGGGT	ACATGACAGATTCCATCTAGCACA
PRODH	TAACGCTGATTCGGGGAGAC	GGGTCCAAACGGCATGTACT
MPAO	CCAGTACCTCAAGACCGACG	GATCTCTCGCACCACCTTGT
amiE	TTCAGCCTTGATCACGTGGG	ACATCGGGCTCAGACTGATTTA

#### 2.3.6 Data analysis

Raw paired-end sequencing reads were first subjected to quality control. Adapter sequences and low-quality bases were trimmed using fastp with parameters: requiring a minimum read length of 20 bp and a mean quality score (Q20) per read of > 20. Since a reference genome for *I. salsoloides* is not available, a *de novo* transcriptome was assembled from the pooled high-quality reads of all samples using Trinity. This assembled transcriptome served as the reference for subsequent analysis. The clean reads from each sample were then aligned back to this *de novo* transcriptome using Bowtie2. Transcript abundance was estimated, and read counts for each gene were generated using RSEM integrated within the Trinity pipeline. Differential expression analysis was performed using the DESeq2 package in R. Genes with an absolute value of log2 fold change (|log2FC| ≥ 1) and a false discovery rate adjusted p-value (FDR< 0.05) were considered significantly differentially expressed (DEGs). Functional enrichment analysis of DEGs was conducted based on the Gene Ontology (GO) and Kyoto Encyclopedia of Genes and Genomes (KEGG) databases. GO enrichment was performed using ClusterProfiler in R, and KEGG pathway enrichment was analyzed with KOBAS, with a significance threshold of FDR< 0.05.

Data were processed using Microsoft Excel 2019 and are presented as the mean ± standard deviation (SD) of three biological replicates. Statistical analyses were performed using SPSS 27.0. Differences among the eight treatment groups were assessed by one-way analysis of variance (ANOVA), followed by Tukey’s HSD *post-hoc* test for multiple comparisons at a significance level of p<0.05. Pearson’s correlation coefficient was used for correlation analysis. Data visualization was conducted using Origin 2022, while omics data analysis and visualization were carried out using the Genepioneer Cloud Platform (http://www.jshycloud.com/) and Chiplot (https://www.chiplot.online/).

## 3 Results

### 3.1 Effects of exogenous GB on seed germination and morphological parameters

**Figure 1:**
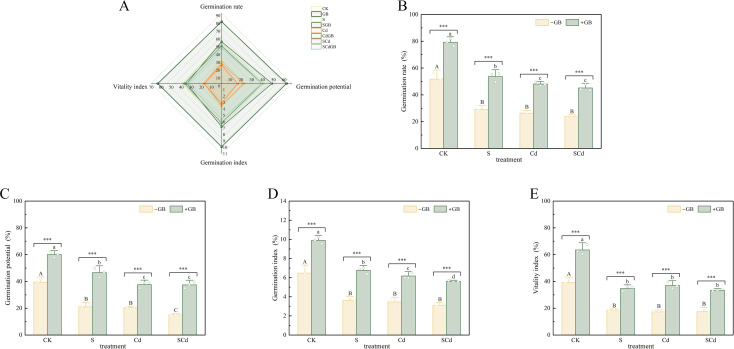
Effects of different treatments on seed germination of *Inula solsoloides***(A)** Radar plot displays the correlations among germination rate, germination potential, germination index, and vigor index. Lines of different colors correspond to different treatment groups; the closer the points on the same axis, the stronger the correlation between the traits. **(B–E)** show the values of germination rate, germination potential, germination index, and vigor index under each treatment group, respectively. Uppercase and lowercase letters indicate significant differences among treatments (p< 0.05) as determined by one-way ANOVA followed by Tukey’s HSD test. Statistical significance is indicated as follows: ***p < 0.001.

Exogenous GB treatment significantly affected seed germination and seedling growth of *I. salsoloides* (**Figure 1**, **Table 2**). GB group significantly enhanced all germination parameters compared to the control (CK), increasing germination rate, germination potential, germination index, and vigor index by 9.89%, 52.19%, 53.01%, and 62.09% (**Figures 1B–E**). In contrast, all stress treatments (S, Cd, SCd) inhibited seed germination. The SCd group showed the most severe suppressive effect, reducing these parameters by 47.15%, 61.82%, 52.09%, and 55.60%, relative to CK. Exogenous GB application alleviated this inhibition. Under combined stress, the SCdGB treatment increased germination rate, germination potential, germination index, and vigor index by 66.91%, 148.64%, 80.97%, and 92.24%, respectively, compared to the SCd group (**Figure 1**). In the SCdGB group, all germination parameters showed substantial recovery, alleviating the SCd-induced inhibition by 75.00% (germination rate), 91.80% (germination potential), 74.48% (germination index), and 73.55% (vigor index), restoring them to 88.21%, 94.93%, 86.71%, and 85.35% of the CK levels, respectively. Thus, GB effectively mitigates the inhibitory effects of combined stress on seed germination.

**Table 2:** Traits of *Inula salsoloides* seedlings under different treatments.

Treatment	Taproot length(mm)		Plant height(mm)		Leaf length(mm)		Leaf width(mm)		Leaf thickness(mm)	
CK	155.64 ± 2.53A	***	60.63 ± 1.17A	*	6.16 ± 0.14A	*	2.37 ± 0.12A	ns	0.61 ± 0.04A	ns
GB	168.61 ± 0.81a		64.10 ± 1.50a		6.51 ± 0.09a		2.48 ± 0.04a		0.65 ± 0.01a	
S	147.57 ± 1.96B	*	51.13 ± 2.42B	ns	4.92 ± 0.27C	ns	1.60 ± 0.30C	ns	0.43 ± 0.05B	ns
SGB	152.35 ± 1.52b		51.42 ± 0.19c		5.21 ± 0.19c		1.74 ± 0.06c		0.46 ± 0.03c	
Cd	141.81 ± 1.80C	ns	50.25 ± 2.20B	**	5.64 ± 0.11B	*	1.94 ± 0.06B	ns	0.56 ± 0.13AB	ns
CdGB	142.35 ± 1.52c		59.96 ± 1.38b		6.01 ± 0.14b		2.06 ± 0.09b		0.60 ± 0.02b	
SCd	130.44 ± 2.31D	**	55.96 ± 3.69A	ns	5.55 ± 0.14B	*	1.81 ± 0.08BC	**	0.57 ± 0.03AB	ns
SCdGB	143.81 ± 4.52c		59.63 ± 2.23b		5.96 ± 0.09b		2.16 ± 0.05b		0.59 ± 0.02b	

Values are mean ± SD (n=3). Different letters within a column indicate significant differences among treatments (p< 0.05, one-way ANOVA, Tukey’s HSD test). Statistical significance is indicated as follows: *p < 0.05, **p < 0.01, ***p < 0.001.

Regarding seedling morphology, GB alone significantly promoted plant growth, increasing taproot length, plant height, leaf length, leaf width, and leaf thickness by 8.33%, 5.72%, 5.68%, 4.64%, and 6.56%, respectively, compared to CK (**Table 2**). Stress treatments inhibited growth through different patterns. Salt stress primarily suppressed leaf development, reducing leaf length, width, and thickness by 20.13%, 32.49%, and 29.51%, respectively. Cd stress mainly inhibited plant height, showing a 17.12% reduction, while combined SCd stress predominantly restricted root growth, decreasing taproot length by 16.19%.

**Figure 2:**
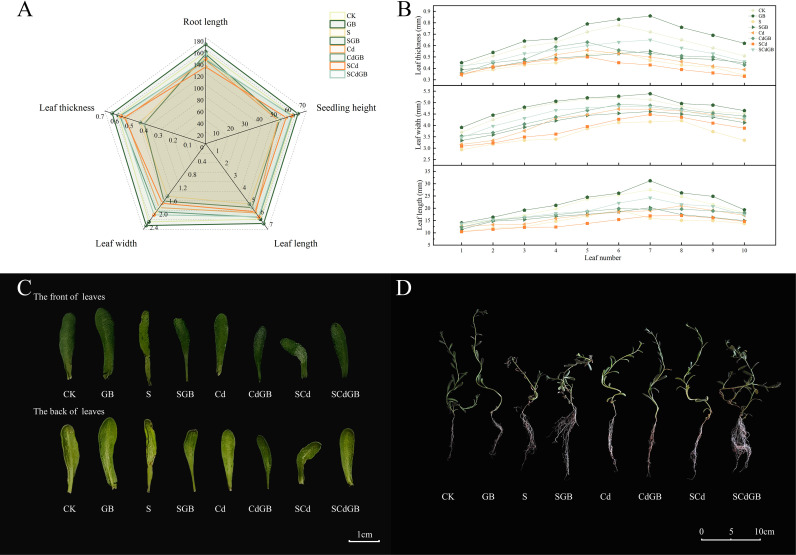
Effects of different treatments on the growth of *Inula salsoloides* seedlings **(A)** Radar plot shows the correlations among root length, seedling height, leaf length, leaf width, and leaf thickness. Lines of different colors correspond to different treatment groups; the closer the points on the same axis, the stronger the correlation between the traits. **(B)** Line chart displays the variation trends in leaf length, leaf width, and leaf thickness from the 1st to the 10th leaf top-down under each treatment group, with different colored lines representing different treatments. **(C)** Phenotypic comparison of the adaxial and abaxial sides of *Inula salsoloides* leaves under different treatments. **(D)** Overall growth morphology of *Inula salsoloides* seedlings under different treatment groups.

Application of GB mitigated these inhibitions. Under salt stress, GB increased taproot length by 3.24%. The CdGB treatment significantly enhanced plant height by 19.32% compared to the Cd group. Under combined SCd stress, GB exhibited particularly prominent effects on taproot length and leaf width, with increases of 10.25% and 19.15%, respectively. Visible stress symptoms such as leaf yellowing and curling were alleviated by GB application (**Figures 2C, D**).

### 3.2 Effects of exogenous GB on physiological and biochemical parameters

**Figure 3:**
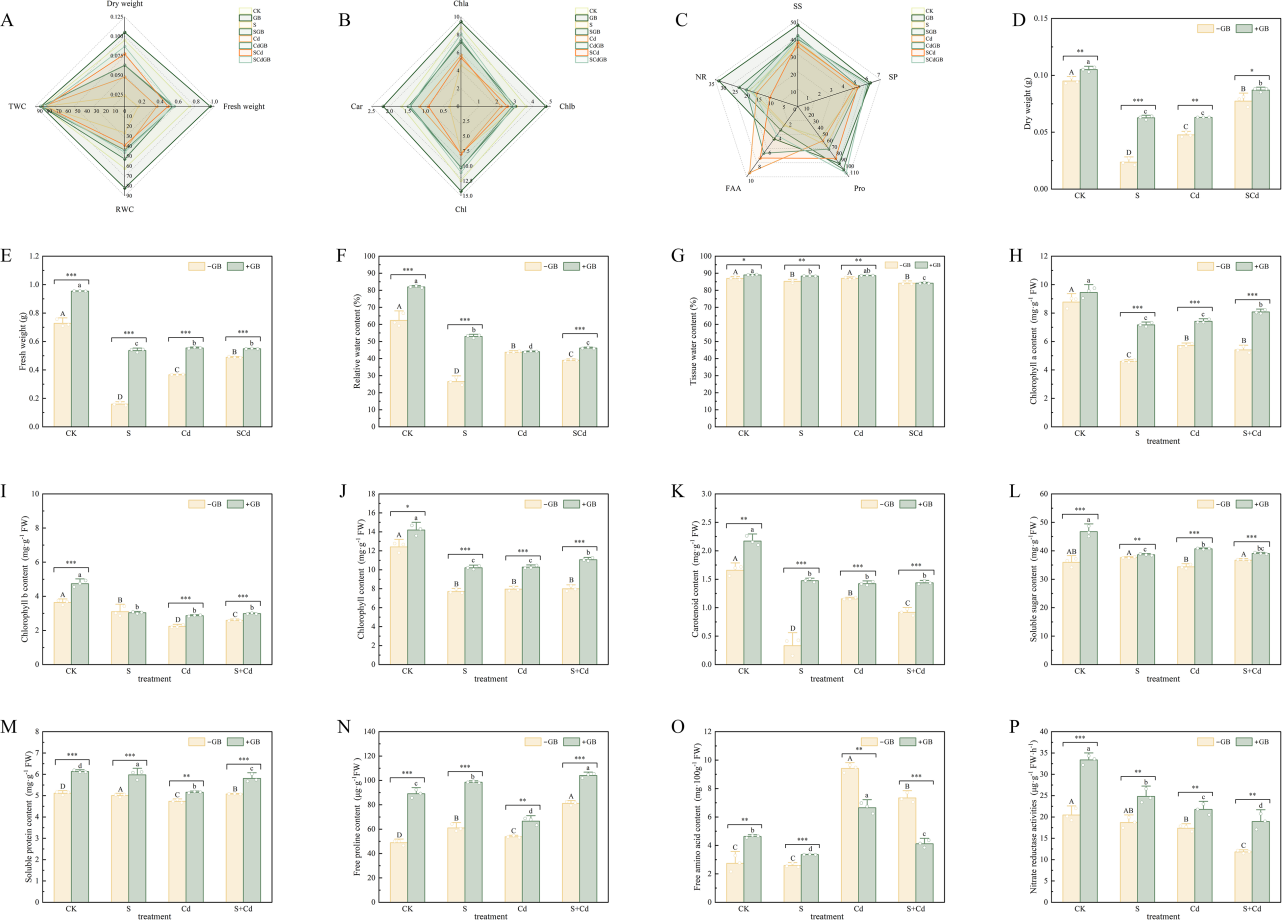
Effects of different treatments on tissue water content, photosynthetic pigments, osmotic adjustment substances, and nitrogen metabolism substances in *Inula salsoloides* seedlings **(A)** Radar plot based on dry weight (DW), fresh weight (FW), relative water content (RWC), and total water content (TWC), reflecting correlations among water-related indices. **(B)** Radar plot based on chlorophyll a (Chl a), chlorophyll b (Chl b), total chlorophyll (Chl), and carotenoid (Car) content, showing associations among photosynthetic pigments. **(C)** Radar plot based on soluble sugar (SS), soluble protein (SP), proline (Pro), free amino acid (AFX), and nitrate reductase (NR), reflecting correlations among osmotic adjustment substances and nitrogen metabolism substances. In all radar plots, lines of different colors represent different treatment groups; the closer the data points on the same axis, the stronger the positive correlation between the two indices. **(D–G)** show the dry weight, fresh weight, relative water content, and total water content of seedlings under each treatment group, respectively. **(H–K)** show the contents of chlorophyll a, chlorophyll b, total chlorophyll, and carotenoids under each treatment group, respectively. **(L–P)** show the contents of soluble sugar, soluble protein, proline, and nitrate reductase under each treatment group, respectively. Uppercase and lowercase letters indicate significant differences among treatments (p< 0.05) as determined by one-way ANOVA followed by Tukey’s HSD test. Statistical significance is indicated as follows: *p < 0.05, **p < 0.01, ***p < 0.001.

**Figure 4:**
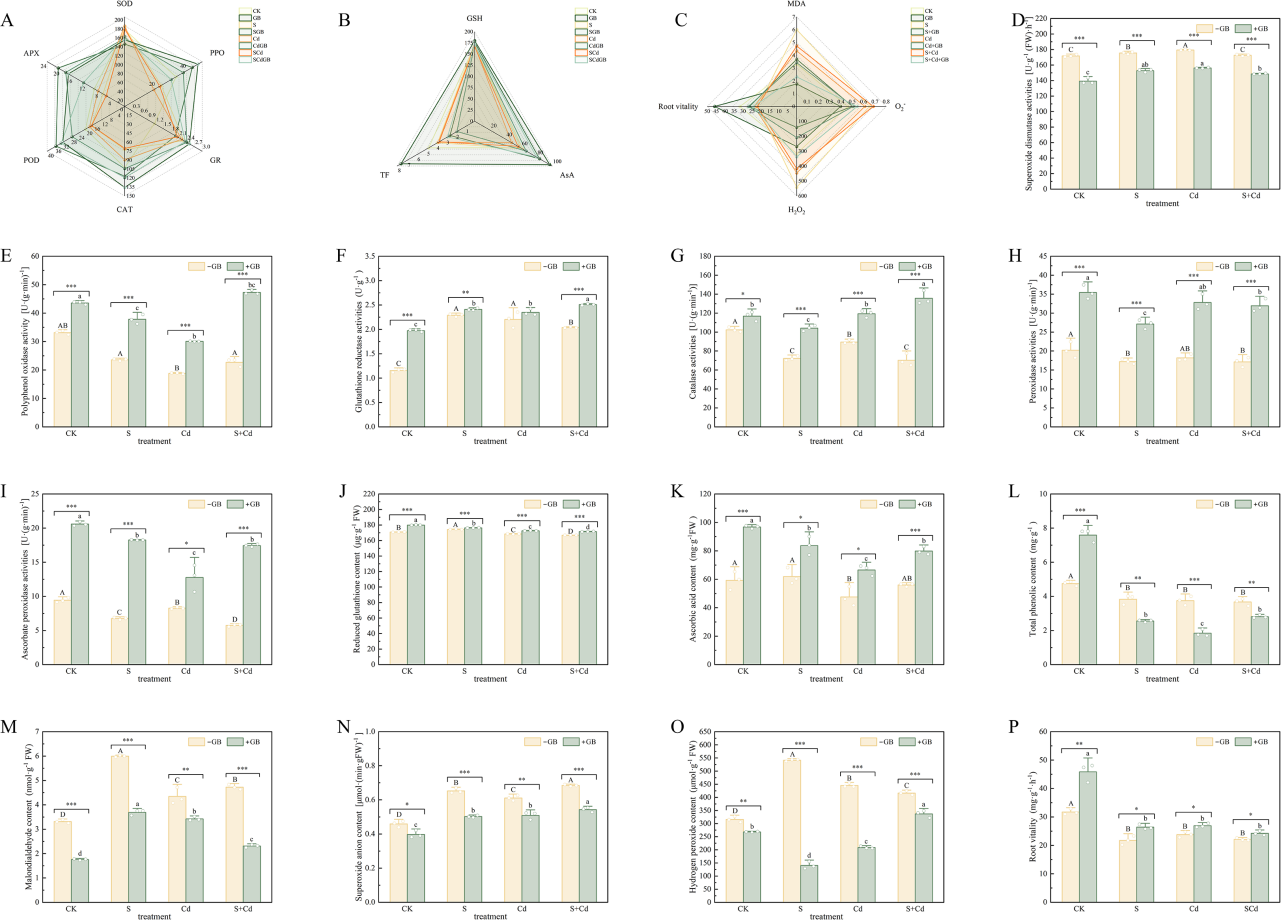
Effects of different treatments on the antioxidant system, ROS accumulation, malondialdehyde content, and root activity in *Inula salsoloides* seedlings **(A)** Radar plot based on the activities of superoxide dismutase (SOD), polyphenol oxidase (PPO), glutathione reductase (GR), catalase (CAT), peroxidase (POD), and ascorbate peroxidase (APX), showing correlations among components of the enzymatic antioxidant system. **(B)** Radar plot based on the contents of reduced glutathione (GSH), ascorbic acid (AsA), and total phenols (TP), displaying associations among non-enzymatic antioxidant substances. **(C)** Radar plot based on the contents of malondialdehyde (MDA), superoxide anion (O_2_^-^), hydrogen peroxide (H_2_O_2_), and root activity, reflecting the correlation between oxidative damage indices and root physiological status. In all radar plots, lines of different colors represent different treatment groups; the closer the data points on the same axis, the stronger the positive correlation between the two indices. **(D–I)** show the activities of SOD, PPO, GR, CAT, POD, and APX under each treatment group, respectively. **(J–L)** show the contents of GSH, AsA, and TP under each treatment group, respectively. **(M–O)** show the contents of MDA, superoxide anion, and hydrogen peroxide under each treatment group, respectively. **(P)** Root activity of each treatment group. Uppercase and lowercase letters indicate significant differences among treatments (p< 0.05) as determined by one-way ANOVA followed by Tukey’s HSD test. Statistical significance is indicated as follows: *p < 0.05, **p < 0.01, ***p < 0.001.

The physiological and biochemical parameters of *I. salsoloides* were significantly altered by stress treatments and modulated by exogenous GB (**Figure 3**, **Figure 4**). Compared to the CK, S, Cd, and SCd stress significantly reduced plant fresh weight (FW) and dry weight (DW) (**Figures 3D, E**). Relative water content (RWC) was also decreased, particularly under Cd stress (**Figure 3F**). GB application improved these parameters under both normal and stress conditions, leading to higher FW, DW, and RWC in the GB-treated groups compared to their corresponding non-GB counterparts (**Figures 3D–G**).

Stress treatments caused a significant decline in the contents of chlorophyll a (Chl a), chlorophyll b (Chl b), total chlorophyll (Chl), and carotenoids (Car) (**Figures 3H–K**). GB application alleviated this reduction, and effectively restored the Car content under combined stress (**Figure 3K**). In response to stress, the contents of osmolytes including soluble sugars (SS), soluble proteins (SP), and proline (Pro) increased (**Figures 3L–N**). The free amino acid (FAA) content showed a marked increase under Cd stress (**Figure 3O**). GB application further elevated the levels of SS, SP, and Pro under most conditions. A prominent effect was observed under Cd stress, where GB (CdGB) reversed the stress-induced accumulation of FAA by 244.69% compared to the Cd-only group (**Figure 3O**).

Regarding the antioxidant system, stress treatments generally suppressed the activities of key enzymes, including superoxide dismutase (SOD), peroxidase (POD), catalase (CAT), and ascorbate peroxidase (APX) (**Figures 4D, G–I**). Concurrently, the levels of oxidative stress markers—malondialdehyde (MDA), superoxide anion (O_2_^-^), and hydrogen peroxide (H_2_O_2_)—were significantly elevated, and root activity was reduced (**Figures 4M–P**). Exogenous GB application counteracted these changes. GB significantly enhanced the activities of antioxidant enzymes, particularly CAT and APX (**Figures 4G, I**), increased the content of non-enzymatic antioxidants such as reduced glutathione (GSH) and ascorbic acid (AsA) (**Figures 4J, K**), and effectively reduced the accumulation of MDA, O_2_^-^, and H_2_O_2_ (**Figures 4M–O**). Root activity was also improved by GB under stress conditions (**Figure 4P**).

### 3.3 Dynamic physiological correlation analysis

**Figure 5:**
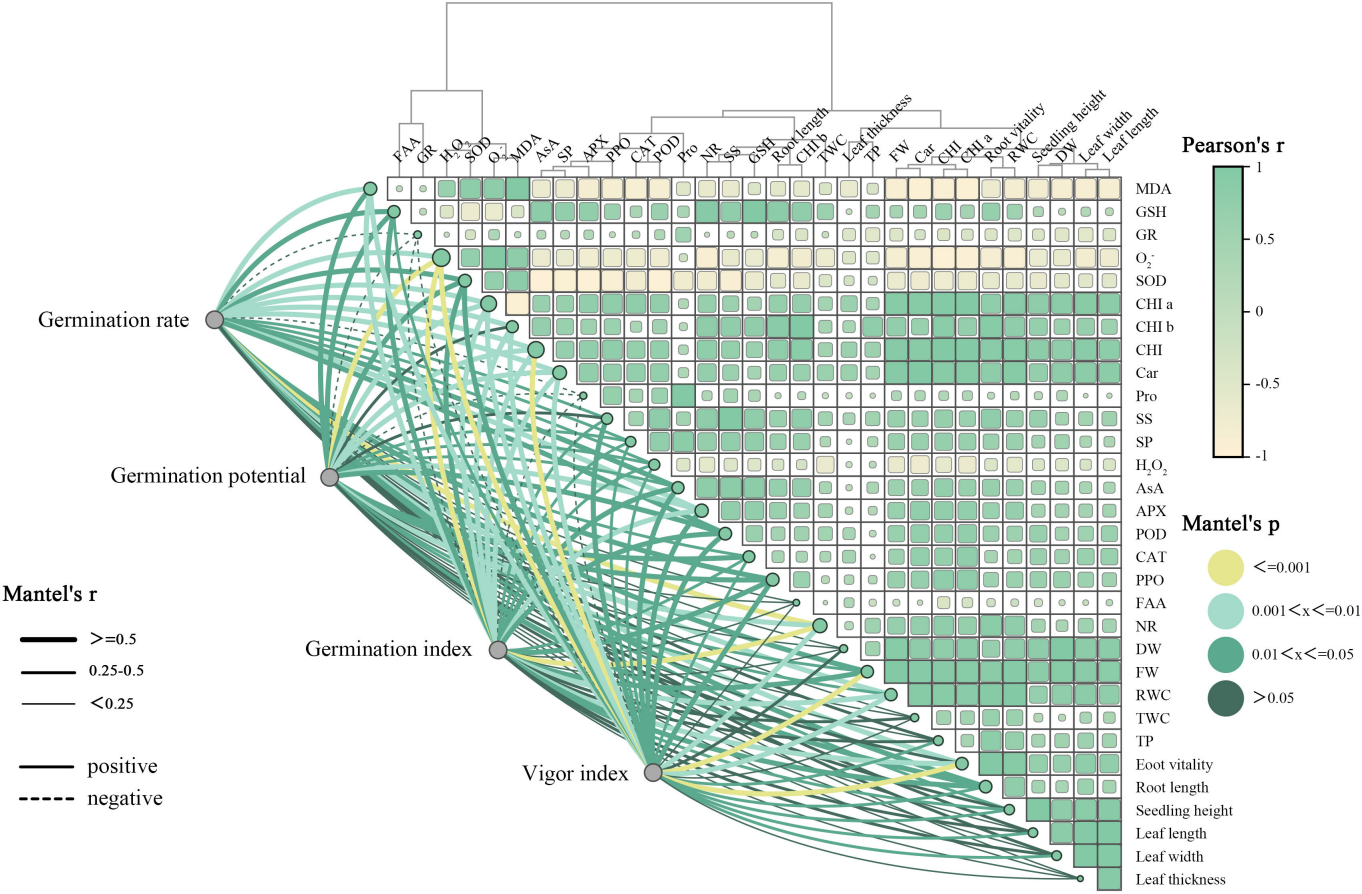
Mantel test and Pearson correlation analysis of dynamic physiological indices in *Inula salsoloides* This figure comprehensively analyzes the correlations between 31 physiological indices and 4 seed germination indices (germination rate, germination potential, germination index, and vigor index) under 8 treatment groups. The left part is a Mantel test network diagram, where nodes represent various measured indices, and connecting lines indicate significant correlations between germination indices and physiological index sets: line thickness is proportional to Mantel’s r value, reflecting the strength of correlation; line color depth reflects the magnitude of Mantel’s p value, indicating the degree of significance; solid lines represent positive correlations, dashed lines represent negative correlations. The right part is a Pearson correlation heatmap, where color depth directly indicates the size of the correlation coefficient between any two variables.

As illustrated in **Figure 5**, significant correlation patterns were observed between the four tested germination parameters (germination rate, germination potential, germination index, and vigor index) and a series of physiological, biochemical, and growth indicators. Antioxidants (GSH, AsA), antioxidant enzymes (GR, APX, POD, CAT), photosynthetic pigments (Chl a, Chl b, Car), osmoregulatory substances (Pro, SS, SP), nitrogen metabolism enzyme (NR), and plant growth parameters (root length, plant height, leaf length, leaf width, leaf thickness, fresh weight, dry weight, root activity, and relative water content) exhibited significantly positive correlations. This pattern demonstrates the synergistic promotion of seed germination by antioxidant capacity, photosynthetic pigment accumulation, osmotic adjustment, and overall growth status. Conversely, oxidative damage markers (MDA, O_2_^-^, H_2_O_2_) showed significantly negative correlations, indicating that ROS accumulation and membrane lipid peroxidation substantially inhibit the seed germination process. These results conclusively demonstrate that seed germination capacity is positively correlated with the plant’s antioxidant defense system and growth vitality, while being negatively correlated with the degree of oxidative damage.

### 3.4 Transcriptome sequencing data statistics

To elucidate the molecular mechanisms underlying GB-mediated stress alleviation, we conducted transcriptome analysis on three independent biological replicates for each of the eight treatment groups. The sequencing generated 1,517,473,406 raw reads. After stringent quality control, which included removal of low-quality sequences, adapter contamination, and ambiguous reads, we obtained 1,509,382,052 clean reads. Quality assessment demonstrated that all samples yielded clean data exceeding 8.15 Gb, with an average GC content of 45.30%. The percentage of Q30 bases was above 95.99% in all samples, and the average Q20 value reached 99.25%, indicating high sequencing quality. The comparable GC content across samples supported subsequent bioinformatic analyses.

*De novo* assembly using Trinity generated 187,331 transcripts and 138,459 unigenes. The assembly demonstrated high completeness, with 40,240 unigenes longer than 1 kb. Unigenes were functionally annotated by conducting BLAST searches against multiple public databases, including the non-redundant (Nr) protein database, Swiss-Prot (Swiss Protein Database), Kyoto Encyclopedia of Genes and Genomes (KEGG), Clusters of Orthologous Groups (COG), Gene Ontology (GO), and Pfam (Protein Family database). A total of 32,049 unigenes were successfully annotated. The Nr database achieved the highest annotation rate (96.57%, 30,950 unigenes), followed by Pfam (90.61%, 29,041), Swiss-Prot (73.42%, 23,529), KEGG (69.10%, 22,146), COG (64.77%, 20,757), and GO (59.28%, 18,999), demonstrating comprehensive database coverage.

### 3.5 Screening of DEGs

**Figure 6:**
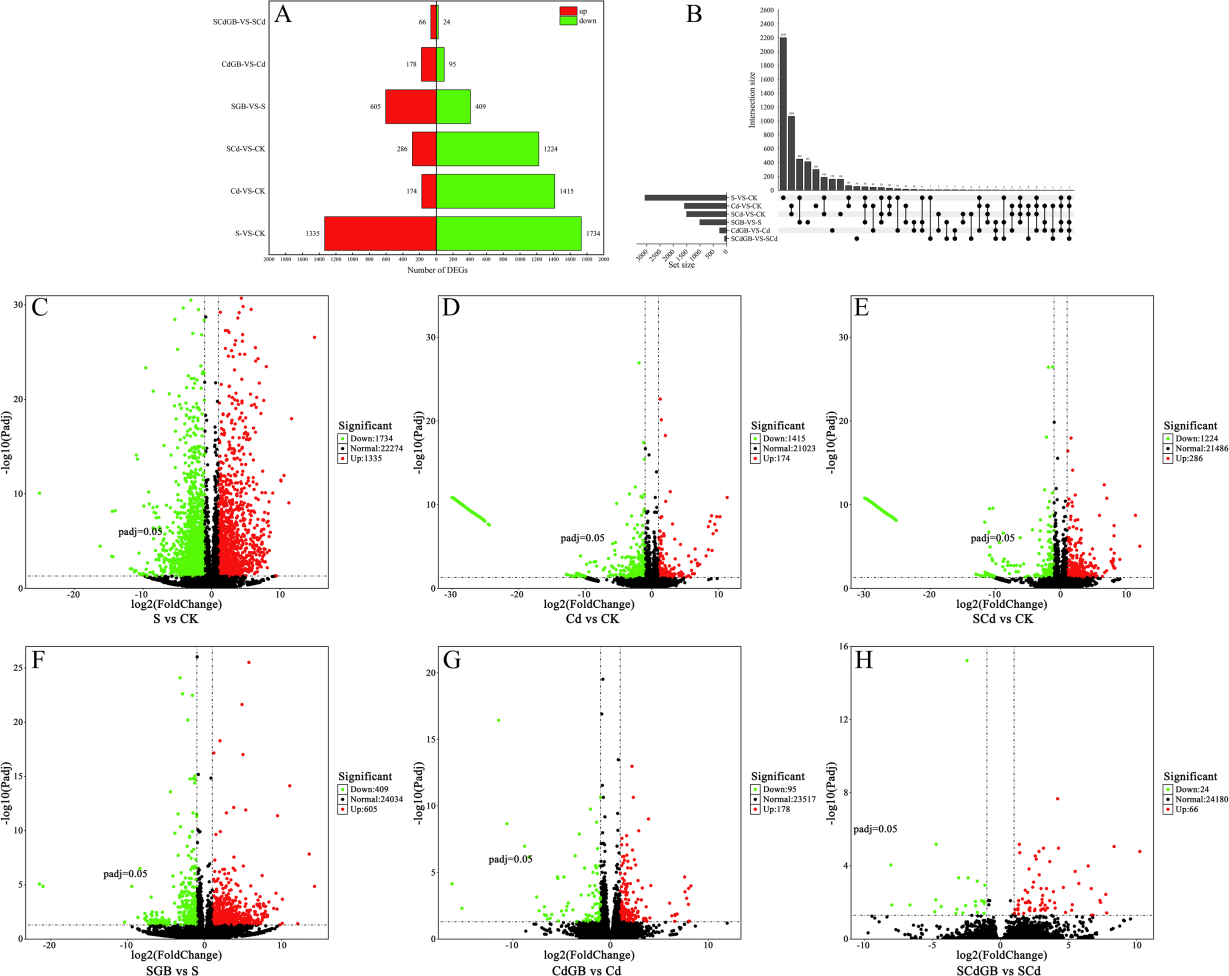
Effects of exogenous glycine betaine on differential gene expression in *Inula salsoloides* seedlings under salt and cadmium stress **(A)** Bar chart of up-regulated and down-regulated significantly DEGs, where red and green bars represent up-regulated and down-regulated genes, respectively. **(B)** Upset plot of DEG sets, showing the number of common and unique DEGs in each comparison combination. **(C–E)** Volcano plots of DEGs for the S-vs-CK, Cd-vs-CK, and SCd-vs-CK comparison groups, respectively, reflecting gene expression changes under single and combined stress. **(F–H)** Volcano plots of DEGs for the SGB-vs-S, CdGB-vs-Cd, and SCdGB-vs-SCd comparison groups, respectively, reflecting the alleviating effect of exogenous GB on the corresponding stress. In all volcano plots **(C–H)**, gray points represent non-significant genes; red points represent significantly up-regulated genes; green points represent significantly down-regulated genes.

To investigate the impact of different stress treatments on the plant transcriptome and the alleviative effect of GB, we performed a systematic analysis of DEGs between each treatment group and the control group. The results (**Figure 6A**) revealed that S, Cd, and SCd stress induced 3,069, 1,589, and 1,510 DEGs, respectively. Specifically, S-vs-CK contained 1,734 down-regulated and 1,335 up-regulated genes; Cd-vs-CK contained 1,415 down-regulated and 174 up-regulated genes; and SCd-vs-CK contained 1,224 down-regulated and 286 up-regulated genes.

Notably, the application of GB modulated the transcriptional response (**Figures 6C–H**): SGB-vs-S yielded 1,014 DEGs (409 down-regulated, 605 up-regulated); CdGB-vs-Cd yielded 273 DEGs (95 down-regulated, 178 up-regulated); and SCdGB-vs-SCd yielded 90 DEGs (24 down-regulated, 66 up-regulated). An Upset plot (**Figure 6B**) illustrating overlaps among comparison groups showed that Cd-vs-CK and SCd-vs-CK shared the highest number of common DEGs (1,066), indicating that the transcriptomic profile under combined stress is largely dominated by the Cd component. Interestingly, the DEG set induced by GB under salt stress (SGB-vs-S) showed only partial overlap with the DEGs altered by salt stress itself (S-vs-CK). This suggests that GB alleviation involves a transcriptional reprogramming that is distinct from, rather than a mere reversion of, the initial stress response signature. Further studies will focus on validating the functions of these shared DEGs and elucidating their associated biological processes.

### 3.6 Enrichment analysis of DEGs

**Figure 7:**
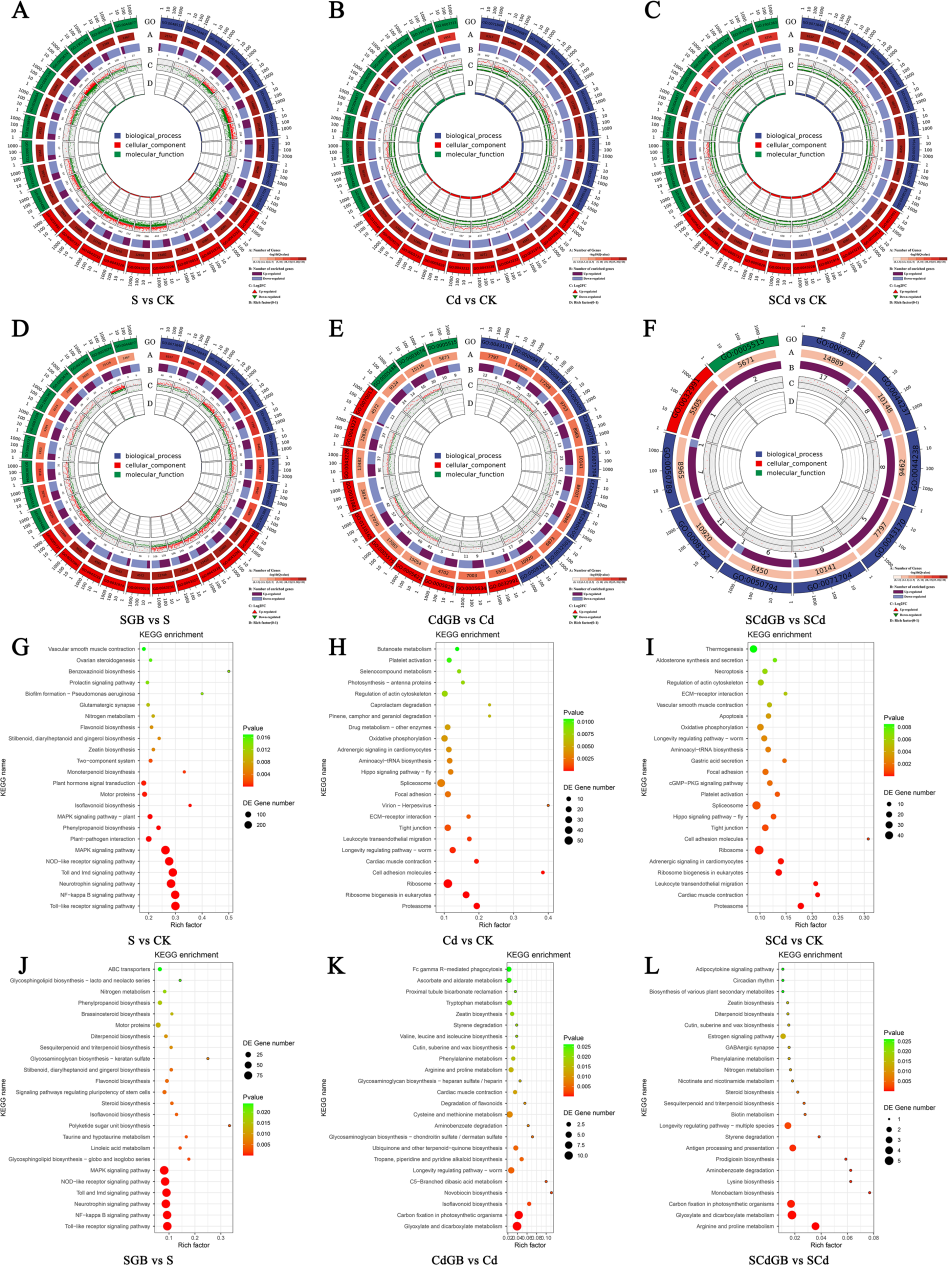
GO and KEGG enrichment analysis of DEGs. **(A–F)** show the GO enrichment diagrams for S-vs-CK, Cd-vs-CK, SCd-vs-CK, SGB-vs-S, CdGB-vs-Cd, and SCdGB-vs-SCd, respectively. As shown in **(A)** the gene number and statistical significance of each significantly enriched GO term are displayed, where bar length represents the gene number and color reflects the enrichment significance (-log_10_(Q value)); **(B)** and **(C)** show the distribution of the number of up- and down-regulated genes in each term and their log_2_(Fold Change) expression level changes, respectively; **(D)** further reveals the enrichment degree of each function through the Rich factor ( the ratio of the number of DEGs in the term to the total number of background genes). **(G–L)** show the KEGG enrichment bubble diagrams for S-vs-CK, Cd-vs-CK, SCd-vs-CK, SGB-vs-S, CdGB-vs-Cd, and SCdGB-vs-SCd, respectively, displaying the top 20 significantly enriched metabolic and signal transduction pathways in the gene set.

GO enrichment analysis revealed that S, Cd, and SCd stresses significantly influenced the DEGs in *I. salsoloides*, displaying distinct enrichment patterns across the three main categories: Molecular Function (MF), Cellular Component (CC), and Biological Process (BP) (**Figures 7A–F**). Under stress conditions, DEGs were consistently and prominently enriched in core biological terms, including “cellular process”, “cellular anatomical entity”, “binding”, and “protein-containing complex”. This pattern underscores that fundamental stress responses primarily involve cellular structure maintenance, basic metabolic regulation, and protein interactions. Notably, the application of exogenous GB markedly altered these enrichment profiles across all stress conditions. In-depth analysis indicated that while GB’s alleviative effects varied depending on the stress type, it consistently and significantly influenced the enrichment of DEGs associated with “cellular process” (GO:0009987), “protein-containing complex” (GO:0032991), and “protein binding” (GO:0005515).

KEGG pathway enrichment analysis further elucidated the alleviative mechanisms of GB from a metabolic perspective (**Figures 7G–L**). During the stress response phase, different stress types exhibited distinct pathway enrichment patterns. Salt stress specifically activated pathways such as “Plant-pathogen interaction”, “MAPK signaling pathway”, and several innate immune signaling pathways (e.g., “Toll-like receptor” and “NF-kappa B signaling pathway”), suggesting a triggered broad stress signal transduction. In contrast, Cd and SCd stress were primarily enriched in pathways like “Ribosome”, “Ribosome biogenesis in eukaryotes”, and “Proteasome”, which is consistent with a severe disruption of protein homeostasis under heavy metal toxicity.

The application of GB induced a significant shift in these stress response patterns. Under salt stress, GB addition did not alter the sustained activation state of the stress signaling pathways, suggesting its alleviative role may operate downstream of or in parallel to these initial signaling events. Most notably, under Cd and combined stress, GB treatment correlated with a fundamental change in the pathway enrichment pattern. The stress-inhibited ribosome-related pathways were no longer significantly enriched, replaced instead by the activation of energy metabolism pathways such as “Carbon fixation in photosynthetic organisms” and “Glyoxylate and dicarboxylate metabolism”. Particularly under combined stress, the enrichment of the “Arginine and proline metabolism” pathway, a crucial pathway for stress adaptation and osmotic adjustment, was especially critical. This study demonstrates that the mechanisms by which GB alleviates S, Cd, and SCd stress in *I. salsoloides* involve both commonalities and specificities. The commonality lies in GB’s core regulation of fundamental functions like “cellular process”, “protein-containing complex”, and “protein binding” to maintain basic cellular activities.

The specificity manifests as: for salt stress which elicits strong signaling stress, GB primarily modulates and orchestrates the defense response; for Cd and combined stress which severely disrupt protein homeostasis, GB effectively reverses toxic damage by remodeling energy metabolism (“carbon fixation in photosynthetic organisms” and “glyoxylate and dicarboxylate metabolism”) and initiating protective synthesis (activating “arginine and proline metabolism” to produce proline), thereby achieving systemic alleviation. This mechanistic insight provides a theoretical basis for the precise application of GB as a plant stress mitigator.

### 3.7 Transcription factors

**Figure 8:**
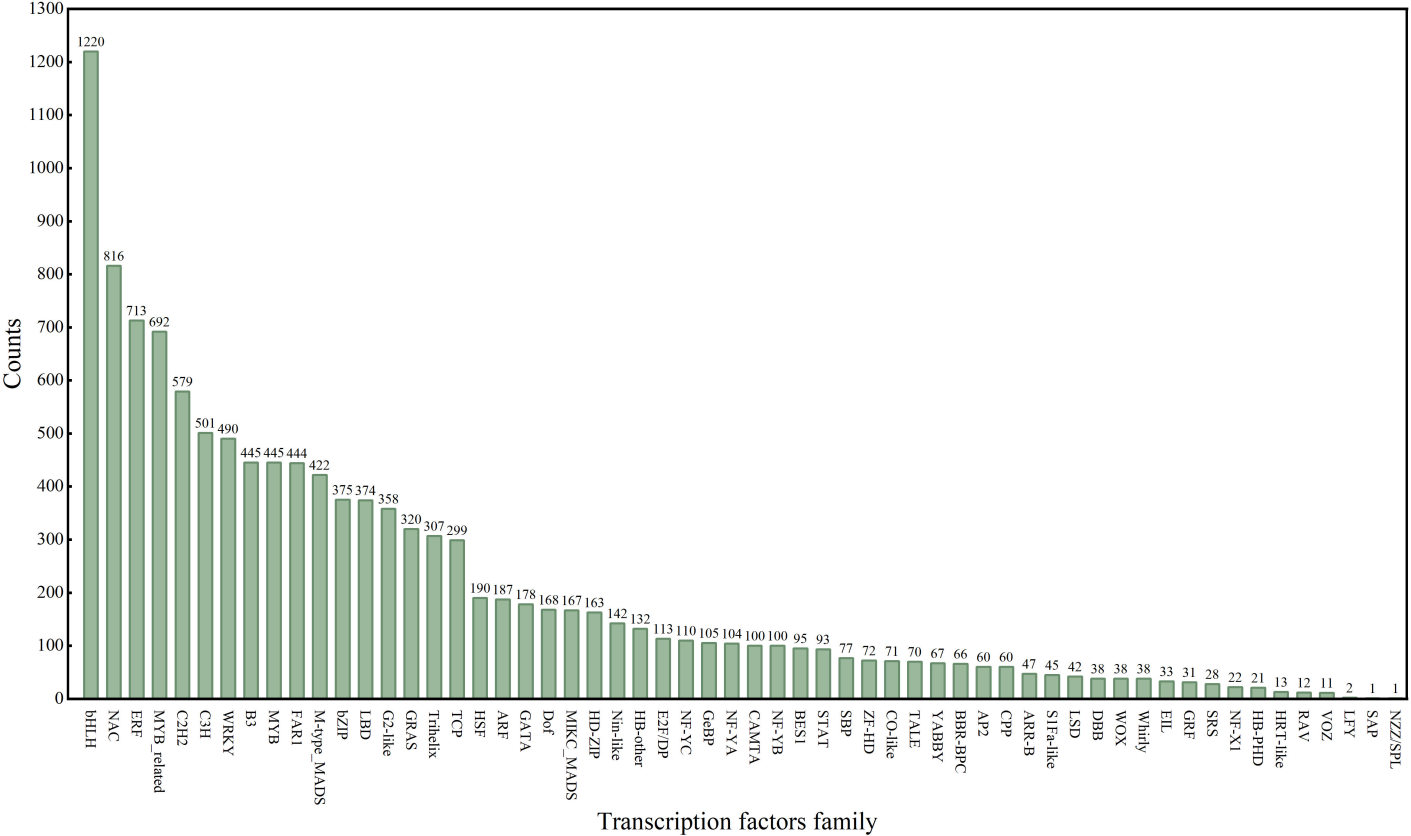
Transcription factor analysis.

A total of 11,913 transcription factors (TFs) were identified in this study, distributed across 58 transcription factor families (**Figure 8**). Among these, eight TF families demonstrated significant associations with S, Cd, and SCd stress responses: bHLH (1,220 genes), NAC (816 genes), ERF (713 genes), MYB-related (692 genes), C2H2 (579 genes), C3H (501 genes), WRKY (490 genes), B3 (445 genes), MYB (445 genes), FAR1 (444 genes), and M-type_MADS (422 genes). The bHLH family represented the most abundant group (19.23%), followed by the NAC family (12.86%). GB application is associated with extensive reprogramming of TF expression, particularly within prominent families such as bHLH and NAC, which are known regulators of abiotic stress responses. This suggests that GB may influence the plant’s stress response network through modulating these key transcriptional regulators; future studies profiling the expression dynamics of specific bHLH and NAC genes under GB treatment will be valuable to confirm their functional roles.

### 3.8 qRT-PCR validation assay

**Figure 9:**
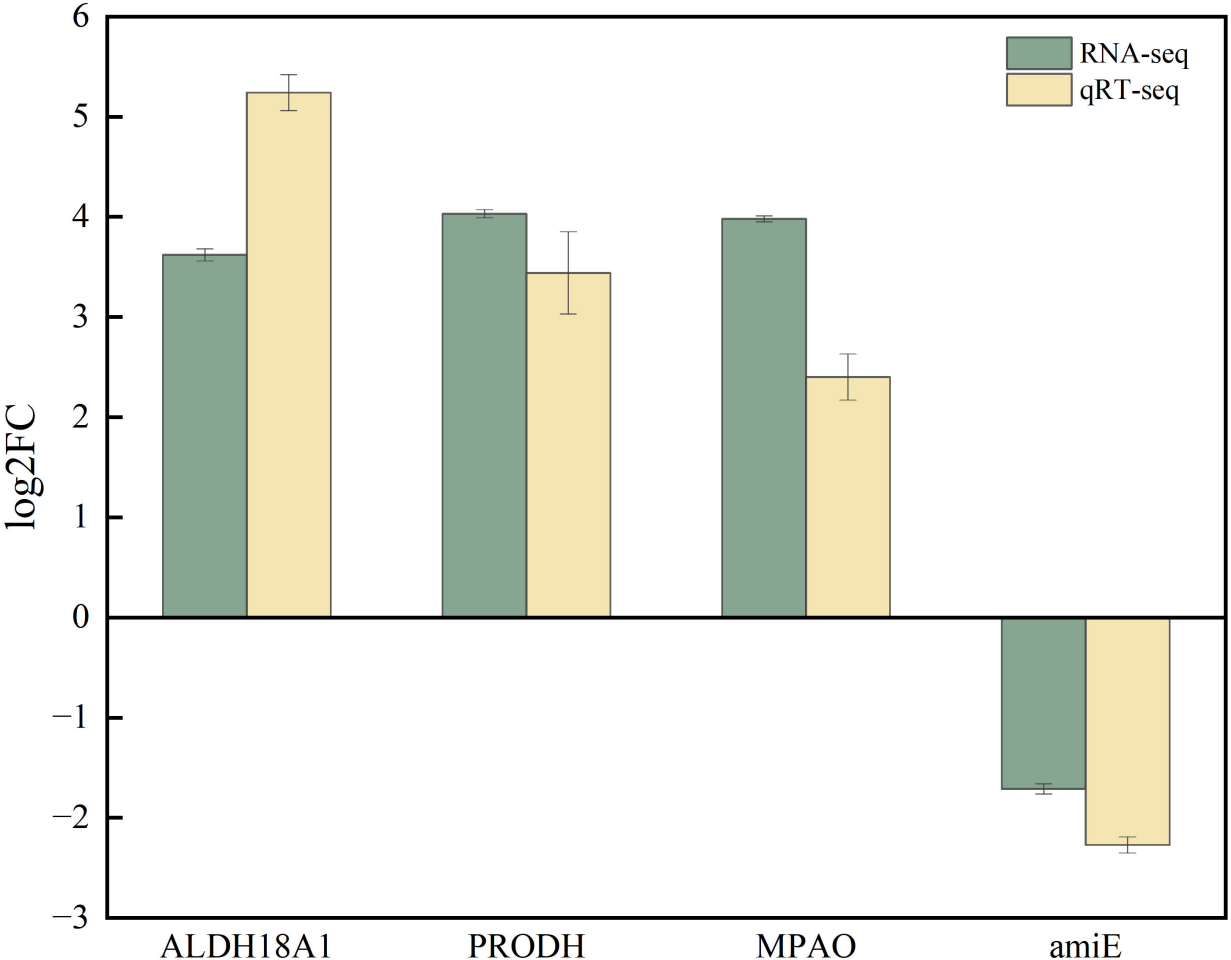
Verification of RNA-seq data by qRT-PCR.

The expression patterns of the four DEGs between the SCd and SCdGB groups were consistent. Among them, three genes—ALDH18A1 (K12657), PRODH (K00318), and MPAO (K13366)—were significantly upregulated, while one gene, amiE (K01426), was downregulated. The expression trends of these DEGs aligned well with the RNA−seq data (**Figure 9**), confirming the high reliability of the transcriptomic results.

## 4 Discussion

Saline-alkali habitats and heavy metal contamination adversely affect plant growth and development, with combined stress often inflicting more severe damage than individual stressors (Fang et al., 2021). This study systematically elucidates the pivotal role of exogenous GB in alleviating S, Cd, and SCd stress during seed germination and early growth of *I. salsoloides*. Our findings, spanning phenotypic, physiological, biochemical, and molecular levels, demonstrate that GB enhances multi-stress tolerance not through a single pathway, but via a synergistic network of interactions.

At the phenotypic level, GB’s promotion of seed germination and seedling growth, along with its restorative effects, constitute the most direct evidence of stress mitigation. GB exhibits a “targeted repair” capability against stress-induced specific growth inhibition patterns—predominantly restoring leaf development under salt stress while focusing on root system recovery under Cd stress. This organ-specific repair pattern aligns closely with plant adaptive response mechanisms: salt stress primarily inhibits leaf expansion through osmotic stress and ion toxicity (Munns and Tester, 2008), whereas Cd stress directly suppresses root elongation by inducing root tip oxidative bursts and disrupting hormone distribution (Wang H. Q, et al., 2020). This indicates that GB’s alleviative effect is stress-type-dependent, functionally compensating for primary damage caused by different stressors. Notably, even under the most inhibitory combined stress, GB substantially enhanced germination potential and vigor index, restoring them to near control levels, underscoring its significant application potential in complex stress scenarios.

Physiologically and biochemically, GB exerts its protective role through three core aspects: maintaining cellular water balance, preserving photosynthetic apparatus integrity, and reestablishing redox homeostasis. GB significantly alleviated stress-induced disruptions in water metabolism and biomass reduction. Moreover, it specifically enhancing key photosynthetic pigments, such as Chl b and Car, thereby safeguarding photosynthetic assimilation. Research indicates that carotenoids function not only as photosynthetic antenna pigments but also as crucial non-enzymatic antioxidants, playing key roles in dissipating excess light energy and scavenging ROS (Havaux, 2014). More importantly, our study reveals GB’s potent regulatory capacity over the plant antioxidant defense system. Under stress conditions, GB not only reversed the suppressed activities of key antioxidant enzymes (e.g., APX, POD, CAT) but also significantly elevated levels of endogenous antioxidants (e.g., GSH, AsA), thereby effectively clearing excessively accumulated H_2_O_2_ and O_2_^-^, ultimately reducing membrane lipid peroxidation product (e.g., MDA) content and protecting cellular membrane integrity. Notably, the activity of NR, the rate-limiting enzyme in nitrate assimilation, was severely suppressed under SCd stress. GB treatment restored NR activity by 71.7%, indicating that GB not only alleviates oxidative and osmotic stress but also mitigates Cd-induced impairment of nitrogen metabolism. This finding aligns with the “antioxidant network” theory proposed by Gill and Tuteja (2010), suggesting that GB may coordinate synergistic interactions among multiple antioxidant components to establish a more efficient oxidative defense system.

The molecular mechanisms underlying these physiological responses were elucidated through transcriptomic analysis. GO enrichment analysis revealed that various stresses and GB treatments significantly impacted core functional categories such as “cellular process”, “protein binding”, and “protein-containing complex”, indicating GB’s potential role in regulating broad cellular activities and protein interactions. Key evidence emerged from KEGG pathway enrichment analysis: under salt stress, GB treatment specifically enriched the “MAPK signaling pathway” and multiple stress/immunity-related signaling pathways, suggesting GB may initiate plant defense programs early by modulating core signal transduction pathways. This finding is supported by Zhu (2016), who emphasized the central role of MAPK cascades in salt stress signaling, with GB potentially participating as a signaling molecule. Under Cd and combined stress, GB treatment shifted metabolic pathways from basic synthesis pathways like “Ribosome” toward “Carbon fixation” and “Glyoxylate and dicarboxylate metabolism”, indicating a reorientation of GB’s strategy toward enhancing energy metabolism and carbon assimilation to provide substantial material and energy resources for stress resistance. This metabolic reprogramming strategy aligns with the “metabolic priority” hypothesis proposed by Clemens (2006), wherein plants reconfigure metabolic fluxes under heavy metal stress to maintain energy supply and carbon skeleton balance. Particularly noteworthy under combined stress was the enrichment of the “Arginine and proline metabolism” pathway, perfectly corroborating our physiological observation of GB-induced proline accumulation, providing molecular-level confirmation that GB finely regulates osmoticum metabolic networks to maintain cellular osmotic balance. This further validates Per et al. (2017) regarding proline metabolism’s central role in plant stress resistance, revealing a novel mechanism where GB enhances osmotic adjustment capacity by upregulating key proline synthesis genes at the transcriptional level.

Exogenous GB contributes to multi-stress alleviation in *I. salsoloides* through combined physiological and transcriptional adjustments. GB functions as an effective osmoprotectant and antioxidant, directly assisting in cellular homeostasis. By activating key signaling pathways, it is associated with the antioxidant defense system, optimizes osmotic adjustment metabolic flux, and enhances photosynthetic carbon assimilation capacity, thereby constructing a more robust stress response system overall. This multi-level, multi-target synergistic mechanism enables GB to effectively combat the complex challenges posed by individual and combined S and Cd stress. Our study provides novel theoretical and experimental evidence elucidating GB’s central role in plant cross-tolerance to abiotic stresses. Exogenous GB alleviates combined stress in *I. salsoloides* primarily through direct physiological protection—acting as an osmoprotectant and antioxidant to mitigate immediate damage. The resultant improvement in cellular homeostasis appears to create a permissive environment that enables the plant’s endogenous stress signaling and transcriptional networks to function more effectively, as evidenced by the observed transcriptomic reprogramming. Thus, GB enhances tolerance via an integrated mechanism of direct protection and facilitation of endogenous adaptive responses.

In parallel, GB treatment is associated with extensive transcriptomic reprogramming, suggesting it may also influence stress-responsive signaling. The coordinated upregulation of antioxidant defenses, osmotic adjustment pathways, and photosynthetic carbon assimilation observed under GB treatment collectively enhances the plant’s capacity to cope with individual and SCd stress. While the exact role of GB as a signaling regulator remains to be fully elucidated, our study provides integrated evidence that GB application establishes a physiological and transcriptional state conducive to stress tolerance.

This study elucidates the protective role of GB under controlled laboratory conditions, which was essential for mechanistic dissection but inherently has limitations. First, our transcriptomic analysis, while revealing broad regulatory patterns, is descriptive; the functional roles of key candidate genes (e.g., specific bHLH or NAC TFs) and pathways require direct validation through techniques like mutagenesis, or transgenic approaches. Second, the dynamic metabolic changes underpinning the observed physiological adjustments remain to be fully characterized. Future research should therefore focus on functional validation of key DEGs and transcription factors identified herein. Furthermore, integrating metabolomic profiling would powerfully complement our transcriptomic data, directly linking gene expression changes to fluxes in key metabolites (e.g., proline, sugars), thereby providing a more complete systems-level understanding of GB’s mode of action.

Our findings strongly support the potential of exogenous GB as a phytoprotectant to enhance plant establishment in saline and heavy metal-contaminated soils. For practical application, key factors such as GB cost-effectiveness, optimal application timing, dosage, and formulation need to be evaluated under scalable field conditions. Pilot trials in actual degraded habitats will be crucial to assess its ecological efficacy and economic viability. If proven successful, GB treatment could become a valuable component in the toolkit for rehabilitating marginal lands and supporting sustainable vegetation recovery.

## 5 Conclusion

This study provides the first systematic elucidation of the mechanisms through which GB alleviates S, Cd, and SCd stress in *I. salsoloides* at both dynamic physiological and transcriptomic levels, offering novel theoretical insights for utilizing xerophytes in ecological restoration and stress tolerance research. Our results demonstrate that S and Cd stress significantly inhibit the growth and development of *I. salsoloides*, impair photosynthesis, and promote ROS accumulation. Application of exogenous GB effectively mitigated stress-induced damage through multiple mechanisms: it promoted the synthesis of osmoprotectants including proline, soluble proteins, and soluble sugars to maintain cellular osmotic balance; simultaneously enhanced the antioxidant defense system by increasing antioxidant content and related enzyme activities, thereby facilitating ROS scavenging and reducing oxidative damage.

Transcriptomic analysis further revealed at the molecular level that GB regulated the expression of numerous stress-responsive genes. Particularly significant was the induction of multiple key TFs by GB, which likely play central regulatory roles in integrating stress signals and activating defense responses. The coordinated responses at both physiological and molecular levels collectively enhance the adaptability of *I. salsoloides* to SCd stress environments. This research provides solid theoretical support for using GB to improve plant abiotic stress resistance and offers a promising technical approach for vegetation restoration in degraded habitats.

## Data Availability

The datasets analyzed during the current study are available in the NCBI repository, National Center of Biotechnology Information under BioProject accession number PRJNA1373009. https://www.ncbi.nlm.nih.gov/bioproject/PRJNA1373009.
