# Supplementary material for: Exogenous glycine betaine alleviates dynamic physiological and transcriptomic responses in Inula salsoloides under combined salt-cadmium stress
Source: Front Plant Sci. 2026 Jan 30;17:1760419. doi: 10.3389/fpls.2026.1760419 (PMC12900682; doi:10.3389/fpls.2026.1760419)
Supplement: Supplementary file 1 [file Table1.docx]

Supplementary Material

# Supplementary Tables

## Supplementary Table 1

Table 1 Statistics of Sequencing Data from *Inula salsoloides* under Different Treatments

| Samples | Total Reads | Clean Reads | Clean bases/bp | GC(%) | Q20(%) | Q30(%) |
| --- | --- | --- | --- | --- | --- | --- |
| CK1 | 60,029,498 | 59,685,906 | 8,984,987,588 | 44.97% | 99.27% | 96.41% |
| CK2 | 62,839,716 | 62,556,534 | 9,405,555,990 | 45.66% | 99.27% | 96.38% |
| CK3 | 64,856,630 | 64,481,308 | 9,703,357,610 | 45.72% | 99.24% | 96.27% |
| GB1 | 60,891,614 | 60,588,714 | 9,115,098,416 | 45.45% | 99.25% | 96.29% |
| GB2 | 67,606,922 | 67,233,860 | 10,115,868,360 | 45.59% | 99.25% | 96.33% |
| GB3 | 66,133,746 | 65,785,998 | 9,898,756,076 | 45.28% | 99.26% | 96.33% |
| S1 | 56,514,964 | 56,236,660 | 8,457,878,572 | 44.74% | 99.27% | 96.36% |
| S2 | 59,638,272 | 59,315,594 | 8,921,111,674 | 44.95% | 99.26% | 96.36% |
| S3 | 62,820,298 | 62,482,064 | 9,390,975,754 | 45.06% | 99.26% | 96.30% |
| SGB1 | 60,082,706 | 59,747,994 | 8,986,207,164 | 44.35% | 99.24% | 96.26% |
| SGB2 | 54,501,212 | 54,199,844 | 8,155,385,158 | 45.28% | 99.25% | 96.27% |
| SGB3 | 76,269,832 | 75,847,738 | 11,412,886,346 | 45.05% | 99.19% | 95.99% |
| Cd1 | 66,533,342 | 66,171,698 | 9,961,406,090 | 45.84% | 99.26% | 96.33% |
| Cd2 | 63,262,186 | 62,909,864 | 9,470,561,754 | 45.74% | 99.24% | 96.25% |
| Cd3 | 68,293,822 | 67,924,814 | 10,225,511,368 | 45.57% | 99.24% | 96.24% |
| CdGB1 | 67,601,682 | 67,264,726 | 10,124,235,440 | 45.36% | 99.27% | 96.40% |
| CdGB2 | 59,882,298 | 59,562,148 | 8,964,299,752 | 45.56% | 99.25% | 96.31% |
| CdGB3 | 61,555,348 | 61,226,068 | 9,211,898,612 | 45.66% | 99.26% | 96.36% |
| SCd1 | 63,005,544 | 62,665,414 | 9,428,636,190 | 45.17% | 99.25% | 96.28% |
| SCd2 | 67,177,800 | 66,820,194 | 10,058,691,494 | 45.60% | 99.27% | 96.39% |
| SCd3 | 63,931,578 | 63,613,790 | 9,575,185,380 | 45.62% | 99.25% | 96.28% |
| SCdGB1 | 65,736,488 | 65,403,496 | 9,837,189,406 | 45.56% | 99.26% | 96.34% |
| SCdGB2 | 57,692,632 | 57,386,184 | 8,634,627,256 | 45.13% | 99.26% | 96.33% |
| SCdGB3 | 60,615,276 | 60,271,442 | 9,074,270,776 | 44.35% | 99.25% | 96.30% |

## Supplementary Table 2

Table 2 Length Distribution Statistics of Sequencing Data for *Inula salsoloides* Samples

| Transcript length interval | 200-500bp | 500-1kbp | 1k-2kbp | >2kbp | Total |
| --- | --- | --- | --- | --- | --- |
| Number of transcripts | 65966 | 37778 | 42424 | 41163 | 187331 |
| Number of unigenes | 65931 | 32288 | 21038 | 19202 | 138459 |

## Supplementary Table 3

Table 3 Statistical Results of Unigenes Function Annotation

| Annotated databases | Gene Number |
| --- | --- |
| Total Unigenes | 32049 |
| Nr | 30950 |
| Swiss-Prot | 23529 |
| KEGG | 22146 |
| gene_ontology_blast | 18999 |
| eggNOG | 20757 |
| Pfam | 29041 |
| CAZy | 2136 |
| Signal | 4442 |
| tmhmm | 6829 |
